# Appraisal of a Contact Tracing Training Program for COVID-19 in Greece Focusing on Vulnerable Populations

**DOI:** 10.3390/ijerph18179257

**Published:** 2021-09-02

**Authors:** Elena Riza, Eleni Kakalou, Evangelia Nitsa, Ioannis Hodges-Mameletzis, Paraskevi Goggolidou, Agis Terzidis, Eleni Cardoso, Karl Philipp Puchner, Zisimos Solomos, Anastasia Pikouli, Eleni-Panagiota Stoupa, Christina Kakalou, Evika Karamagioli, Emmanouil Pikoulis

**Affiliations:** 1 Department of Hygiene, Epidemiology & Medical Statistics, Medical School, National and Kapodistrian University of Athens, Mikras Asias 75, 11527 Athens, Greece; eriza@med.uoa.gr (E.R.); enitsa@med.uoa.gr (E.N.); 2 Postgraduate Programme “Global Health-Disaster Medicine”, Medical School National and Kapodistrian University of Athens, Dilou 1 Street, 11527 Athens, Greece; ekakalou@yahoo.gr (E.K.); yanni.hodges@gmail.com (I.H.-M.); agisterzidis@gmail.com (A.T.); cardosoeleni@gmail.com (E.C.); karl.puchner@gmx.de (K.P.P.); crisismed@outlook.com (A.P.); eldastoupa@hotmail.com (E.-P.S.); ckakalou@gmail.com (C.K.); mpikoul@med.uoa.gr (E.P.); 3 Faculty of Science and Engineering, University of Wolverhampton, Wolverhampton WV1 1LY, UK; P.Goggolidou@wlv.ac.uk; 4 Hellenic Red Cross, Dilou 1 Street, 11527 Athens, Greece; zisimos31884@yahoo.com

**Keywords:** COVID-19, contact tracing, training course, vulnerable groups, communication skills, evaluation

## Abstract

Background: Contact tracing as an epidemiological strategy has repeatedly contributed to the containment of various past epidemics and succeeded in controlling the spread of disease in the community. Systematic training of contact tracers is crucial in ensuring the effectiveness of epidemic containment. Methods: An intensive training course was offered to 216 health and other professionals who work with vulnerable population groups, such as Roma, refugees, and migrants in Greece, by the scientific team of the postgraduate programme “Global Health-Disaster Medicine” of the Medical School, National and Kapodistrian University of Athens, with the support of the Swiss embassy in Greece. The course was delivered online due to the pandemic restriction measures and was comprised of 16 h over 2 days. The course curriculum was adapted in Greek using, upon agreement, a similar training course to what was developed by the Johns Hopkins University Bloomberg School of Public Health. Evaluation of the course was conducted in order to determine the short term satisfaction from participating in this training course. Results: A total of 70% of the course participants completed the evaluation questionnaires and all trainers gave feedback on the course. The training modules were ranked as extremely useful by the majority of the participants and over 50% of the participants specifically stated that the course content was directly related to their work with vulnerable groups. Content about the ethics of contact tracing and the effective communication skills presented were deemed most useful. Conclusion: The course was well organised and provided the required skills for effective contact tracing. Many course participants intend to use some components in their work with vulnerable populations groups. Contact tracing efforts work best in a systematic and coordinated way and the provision of systematic and organised training can greatly increase its effectiveness.

## 1. Introduction

### The Current-Status of COVID-19 Contact Tracing in Greece

According to the Greek National Public Health Organization (NPHO), the first confirmed case of COVID-19 in the country was reported on 26 February 2020 [1]. Closure of schools, universities, shops and other facilities was gradually implemented, until a restriction of movement was enforced throughout the country [2]. By the end of March 2020, a total of 1,212 confirmed cases and 46 deaths were reported. Over a year later, on 11 June 2021, the number of confirmed cases was 413,170 and a total of 12,370 deaths had been recorded [3].

Contact tracing activities were initiated and implemented by the NPHO, as part of a public health response intended to reduce further spread of COVID-19. Health professionals were recruited to offer contact tracing services, via telephone communication with confirmed cases. This service was critical in order to trace any possible COVID-19 cases, as well as to provide information and guidance to both positive cases and close contacts. The NPHO followed a specific protocol, where for every laboratory-confirmed SARS-CoV-2 positive result, the closest contacts had to be identified and tested. If a contact among those tested was found positive, his/her closest contacts had to be identified and tested as well [4].

Confirmed cases were asked to self-isolate at home, and monitor for symptoms, and upon further progression of symptoms to visit a hospital. A communication routine was established, all through the isolation period (10 days, according to guidelines by the European Centre for Disease Prevention and Control (ECDC)), in-order to follow up with patients and record the severity or onset of symptoms. “Household contacts” were instructed to quarantine for 14 days, and to monitor for any symptoms and to avoid any social interactions or travel during this period.

The national guidelines of the NPHO have been adjusted according to the guidelines outlined by the ECDC with regards to the monitoring of the pandemic, the definition of confirmed or suspected cases, the transmission pathway and timeline, and instructions to citizens. As the epidemic in Greece unfolded, the Contact Tracing Service became the responsibility of the General Secretariat for Civil Protection (GSCP). The recruitment of 192 contact tracers, located in many areas throughout Greece, was announced in November 2020, with a contract period of eight months and possibility for extension. A basic training was provided for the tracers, with no specific university degree required [5]. To the best of our knowledge, this training delivered by the public health authorities did not take into account the specific needs of hard-to-reach population groups, particularly refugees, migrants, and the Roma communities residing in Greece. However, it has not yet been possible to confirm the implementation or possible outcomes of this contact tracing activity.

Contact tracing, as an epidemiological strategy, has historically contributed to the containment of past epidemics. For example, contact tracing was a critical intervention in the management of outbreaks of sexually transmitted infections (STIs), Ebola, and SARS [6]. In the context of COVID-19, countries that rapidly implemented and scaled-up tracing efforts, such as Vietnam, Taiwan, New Zealand, Australia and South Korea, were able to successful limit transmission [7]. Nevertheless, effective contact tracing requires skills from public health professionals that can only be acquired through uniform and standardised high-quality training [8]. Such organised efforts contribute to promoting public health interventions, as has already been shown with screening for infectious diseases in newly arrived migrants [9] In the midst of the COVID-19 pandemic response in Greece, it became clear that the contact tracing needs of hard-to-reach population groups were not adequately considered or decisively incorporated in any published national strategic planning, opening an area of academic public health intervention.

Evidently, the COVID-19 pandemic has produced adverse effects on most aspects of human life. The implementation of structured contact tracing activities is of paramount importance in order to mitigate the pandemic effects, such as the deterioration of mental health with conditions including depression and anxiety, changes in eating behaviours [10,11] or even implications in accommodation decision-making [12]. Moreover, the pandemic has had a marked negative impact on specific population groups, such as chronic disease patients [13] and university students [14,15].

In an effort to advocate for effective contact tracing for COVID-19 case contacts in vulnerable population groups in Greece, the scientific team of the postgraduate program of Global Health- Disaster Medicine (Medical School National and Kapodistrian University of Athens-NKUA), with the financial support of the Swiss Agency for Development and Cooperation (SDC), under the project ‘Greece, Strengthening of the Greek Health System (Corona Virus/COVID-19)’ with PR. NO. 7F-10589.01 launched a two day structured training program for professionals who could be incorporated into the SARS-CoV-2 national contact tracing activities in the country, as their training has put emphasis on the specific needs of vulnerable and hard-to-reach populations. Evaluation of the course was conducted to determine the short-term satisfaction from participating in this training course. Findings from the evaluation would support the optimisation of contact tracing activities in vulnerable population groups and to incorporate suggestions into the national epidemiological surveillance and pandemic response plan.

To our knowledge, there is no formal evaluation report that is publicly available for the Greek government contact tracing programme. Key performance indicators (KPIs) on contact tracing, categorised by prefecture (region), in Greece could be informative for the broader research community. In addition, we would advocate for such publicly available reporting, as it would further build trust in contact tracing activities for the Greek public. Some limited information is available on the online COVID-19 policy portal maintained by WHO European Office [16]. 

Greece adopted the ECDC policy for contact tracing, but no dedicated website to contact tracing or a dashboard was available as is the case in other countries.

## 2. Materials and Methods

The content of the contact tracing training program was based on an open online course developed by the Bloomberg School of Public Health of Johns Hopkins University (JHU BSPH) [17]. The scientific committee of the NKUA’s postgraduate program was in contact with and advised by colleagues at JHU BSPH concerning the content implementation of their online course. The Greek team modified the contents of the course to better fit to the local conditions and to the characteristics of the target audience by adapting case scenarios, information on resources for medical care and social support. The course was advertised to health and other professionals with some degree of experience in working with vulnerable population groups. As these groups (Roma, refugees and migrants and solitary people in the community) are recognised for their inherent vulnerability, our intention was to train a group of professionals in contact tracing who would be sensitised to the challenges of working with such populations [18,19].

As such, we contacted professionals working in the healthcare system (primary healthcare units, hospitals), in municipalities, in non-governmental organisations (NGOs) and in Reception and Identification Centres (RICs) that house refugees in Greece. An invitation letter was sent stating the main aims of the training intervention. Upon acceptance to the course, a signed consent form was requested from each participant. In total, two training sessions were conducted online, due to the COVID-19 restriction measures. Each session included five different training modules presented by several instructors over two days, with a total of 16 training hours. Each module contained baseline information on each topic presented, a short quiz, and discussion with the instructors and other participants. The first training session was organised from 19th to 20th December 2020, while the second training session was implemented from 9th to 10th January 2021. All participants (216 in total) were present during the whole duration of the course, and attendance records were kept by the course’s secretariat.

In-order to gain a better insight into the training activity, we developed a questionnaire to be used as part of a quantitative evaluation methodology to assess the appropriateness, effectiveness, level of satisfaction and sustainability of the program. Some qualitative elements were also used to capture the opinions, attitudes towards both the training and contact tracing as a public health strategy, and perceptions for future activities among course participants. Moreover, a short qualitative interview was performed with each course instructor on their experience delivering the course.

In this regard, we formed three different outcome/impact indicators to summarise the impact/performance of the training course:

- Awareness and knowledge of the training course participants: the level of information received, including cultural awareness and sensitivity of the prospective contact tracers after the training.
- Satisfaction of training course participants: the level of meeting or surpassing the expectations and needs from the training course and its related activities.
- Potential to create opportunities for enhancement of the contact tracing network in Greece: the degree of usability of the course material in the trained participants’ work settings, taking into account their work experience.

## 3. Results

Following the completion of the course, an anonymous online questionnaire was sent to all participants. A total of 112 responses were received and analysed, which corresponds to a response rate of 51.8% of the total participants.

The questionnaire consisted of four sections: Section 1 provides general demographic, professional background and employment data; Section 2 records the evaluation of each module of the contact tracing course; Section 3 allows participants express their views on prospects for the future and use of the course; and in Section 4 the participants express their views on the strongest elements of the course and have the opportunity to suggest points for improvement.

### 3.1. Section 1 General Information

The majority of the responding course participants belonged to the 25–34 (38.4%) and 35–44 (37.5%) age groups followed by the 45–54 (16.1%) age group. Most of the respondents were female, at 77.7% (87), and 22.3% (25) were males.

A total of 27.7% of participants (31) were employed in hospitals, 17.9% (20) in NGOs, 44.6% (50) in Primary Health Care units and municipalities, and 9.8% (11) in reception and identification refugee Camps. Regarding their working experiences, 54.4% were employed in their current position for up to three years, whereas 45.5% were employed for over three years.

### 3.2. Section 2A Evaluation of the Contract Tracing Modules

MODULE 1: **Basics of COVID-19**: the following areas were covered:

- Description of the origins of the virus that causes COVID-19;
- Identification of the clinical signs and symptoms of COVID-19 and risk factors for severe disease;
- Description of COVID-19 diagnostic criteria, natural history of the disease, risk factors for severe disease;
- Description of the incubation period, infectious period;
- Explanation of virus person-to-person transmission, and;
- Basic knowledge on disease testing, types of tests available.

A total of 71.4% of participants found the module to be extremely useful for their work and 18.8% of participants found the module to be somewhat useful, whereas 60.7% of participants totally agreed and 31.3% of participants somewhat agreed that the information presented was of very high quality. Thirty-eight participants (33.9%) stated that the module was directly relevant to their work and 29 participants (25.9%) stated that the module was somewhat related. The way that the presentations were delivered by the trainers was totally satisfactory for 72.3% (81) of participants and somewhat satisfactory for 25% (28) of participants.

**Figure 1:**
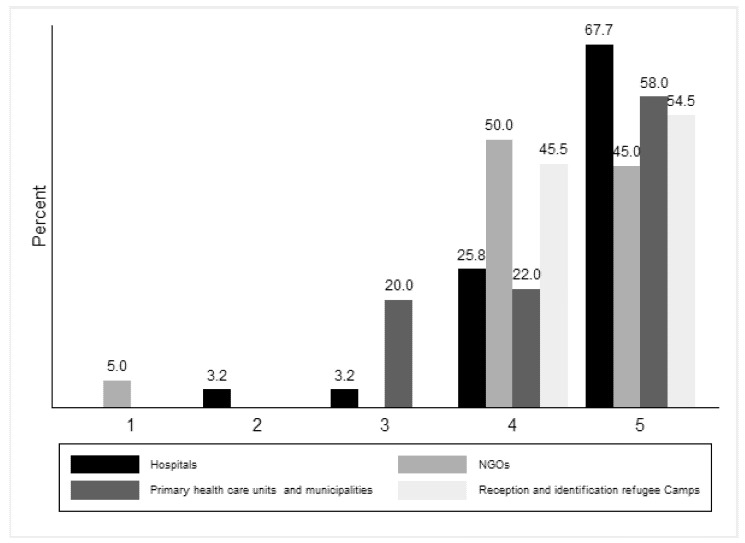
For Module 1 degree of agreement with the phrase “Presents the situation in a complete way” by work experience and profile.

The participants’ opinion on the statement “Presents the situation in a complete way” differed according to their work experience (p_fisher’s_ = 0.02, graph 1). Figure 1 details the results of participants’ opinion on “Presents the situation in a complete way” by work experience and profile.

MODULE 2: **Contact tracing for COVID-19 prevention:** the following areas were covered:

▶ Describe what contact tracing is and how it stops transmission of SARS-CoV-2;
▶ Define a case of COVID-19 and a contact;
▶ Explain the meaning and purpose of isolation and quarantine;
▶ Calculate how long a case should isolate and how long a contact should quarantine;
▶ Describe the connection between the infectious period and isolation and quarantine;
▶ Identify high-risk settings for transmission that require extra action.

In Module 2, 60 participants (53.6%) totally agreed that the information presented was directly related to their work with migrants or refugees and other vulnerable population groups, whereas 29 persons (25.9%) somewhat agreed. The majority of participants (63.4%) stated that the information was of very high quality and 63.6% of participants found the presentation to be very clear. The type of work experience of the participants did not affect this judgement.

MODULE 3: Steps to investigate cases and trace contacts: the following areas were covered:

▶ Identify all the steps to investigate cases and trace their contacts;
▶ Provide examples of the kinds of questions you might ask at each step;
▶ Describe the kinds of social support that cases and contacts may need to carry out isolation and quarantine;
▶ Present an example of a simple case investigation and contact tracing call.

In Module 3, 59.8% (67 respondents) stated that the information presented gave a very clear overview of the contact tracing process (totally agree) and 32.1% (36 persons) somehow agreed with this statement. In similarity to the previous two modules, the information presented was of very high quality (59.8% totally agree and 34.8% somewhat agree) and the presenters were very efficient (70.5% totally agree and 24.1% somewhat agree).

**Figure 2:**
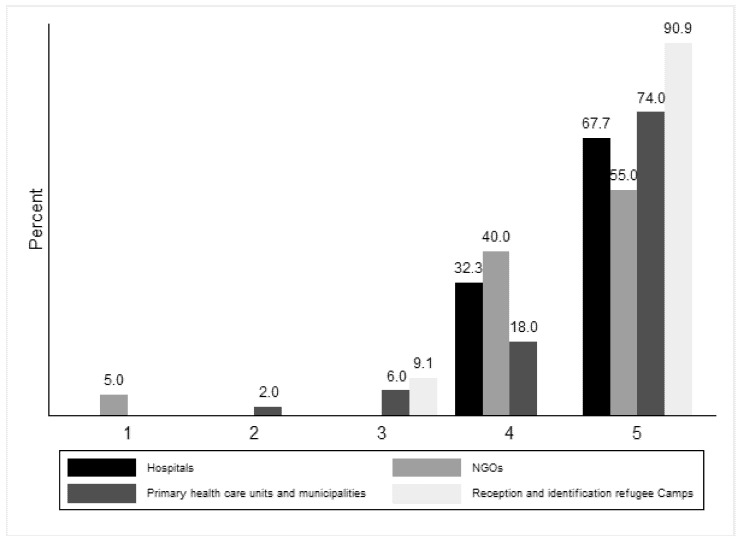
For Module 3, degree of agreement with the phrase “The speaker’s presentation was clear” by work experience and profile.

The participants’ opinions on the statement “The speaker’s presentation was clear” differs depending on the type of work experience of the participant (p_fisher’s_ = 0.056, Figure 2).

MODULE 4: **Ethics of contact tracing:** the following areas were covered:

▶ Define and provide examples of important terms, such as *privacy*, *autonomy*, and *public good*;
▶ Describe the balance between protecting public health and limits to privacy and autonomy;
▶ Provide examples of the balance between keeping information private and protecting public health;
▶ Identify a selection of technological tools that have been developed or used for each step of case investigation and contact tracing.

This Module was well appreciated, and it sparked very interesting questions and discussion. An estimated 53.6% of respondents (60 persons) stated that the information in this module will be directly relevant to their work, while 30.4% of the respondents (34 persons) mentioned that they already work with refugees/migrants and other vulnerable population groups. Sixty-eight of the respondents found that the information on ethics was of a high quality (60.7%) and 66.1% (74) stated that the trainers were very clear and to the point.

MODULE 5: **Skills for effective communication:** the following areas were covered:

▶ Describe the meaning and importance of rapport (mutual understanding, trust, and agreeableness);
▶ Explain ways to an effective communication with cases and contacts;
▶ Understand the difference between question types (open versus closed);
▶ Describe what ‘’active listening’’ means;
▶ Explain the types of human communication and how they apply to contact tracing (active listening, empathy, honesty, reassurance);
▶ Describe and troubleshoot common difficulties with case investigation and contact tracing.

Module 5 was also very well received by the course participants, as it presented communication skills in health-related matters, an area which is not effectively covered in most undergraduate course curricula in Greece. An estimated 64.3% of participants totally agreed that these skills are related with their work and 26.8% somewhat agreed; in addition, 40% of participants declared that this information is directly applicable to their work with refugees or migrants and other vulnerable population groups. In total, 104 participants (92.9%) responded that the information presented was of a very high quality and 107 (95.6%) stated that the presenter was very thorough and clear.

### 3.3. Section 2B: Effectiveness and Knowledge Acquisition

In this section, the contact tracing course participants were asked to rank the degree in which each module helped them improve their knowledge, skills and competence in the areas covered in each module.

MODULE 1: An estimated 72.4% of participants stated that the module had a high degree of effectiveness and helped their skill competencies.

MODULE 2: With regard to the contact tracing steps for COVID-19, 95 participants (84.8%) stated that the module increased their knowledge and skills and helped them to clarify several definitions, such as the difference between quarantine and isolation.

MODULE 3: Regarding the steps of contact tracing outlined in this module, a total of 81.3% (91 respondents) stated that their knowledge and skills increased significantly.

MODULE 4: Concerning the ethics around contact tracing, 84% (94) of participants ranked the acquisition of new knowledge and competencies as very high.

MODULE 5: The communication skills presented in this module were highly appreciated by a total of 89 respondents (79.4%).

### 3.4. Section 3: Level of Satisfaction, of Sustainability and Potential of the Course to Contribute to the Control of the Pandemic in Greece

Through the responses of the course participants to the evaluation questionnaire it was found that:

The course participants (*n* = 96, 85.7%) improved their understanding of COVID-19 throughout the course and gained insight into specific issues of interest with regard to contact tracing efforts among migrants/refugees/vulnerable groups.

An estimated 70.6% (79 participants) stated that the course has improved their communication skills and competencies, especially regarding the difficulties refugees, migrants and other vulnerable groups face in their contact with the healthcare system.

The satisfaction from attending the course and the potential of delivery in colleagues and other working environments was evident and 91 of the participants (81.3%) declared that they would like to receive further training on issues related to the management of the COVID-19 pandemic and that they intend to suggest this course to their colleagues.

According to the respondents, the course has the potential to contribute to the successful control of the pandemic in Greece (98 participants, 87.5%).

**Figure 3:**
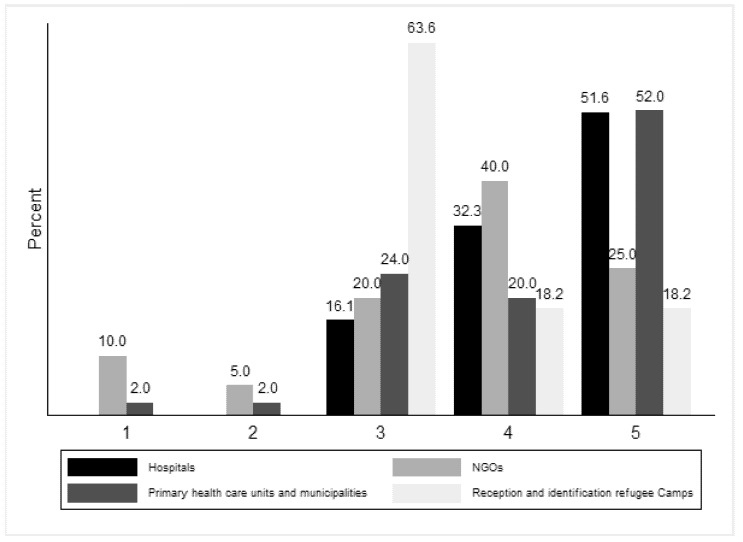
Degree of agreement with phrase “The training course gave me a new insight into the difficulties of immigrants/refugees during the pandemic” by type of work experience and profile.

The participants’ response to the statement “The training course gave me a new insight into the difficulties of immigrants/refugees during the pandemic” differed depending on the work experience of the participant (p_fisher’s_ = 0.03, Figure 3).

**Figure 4:**
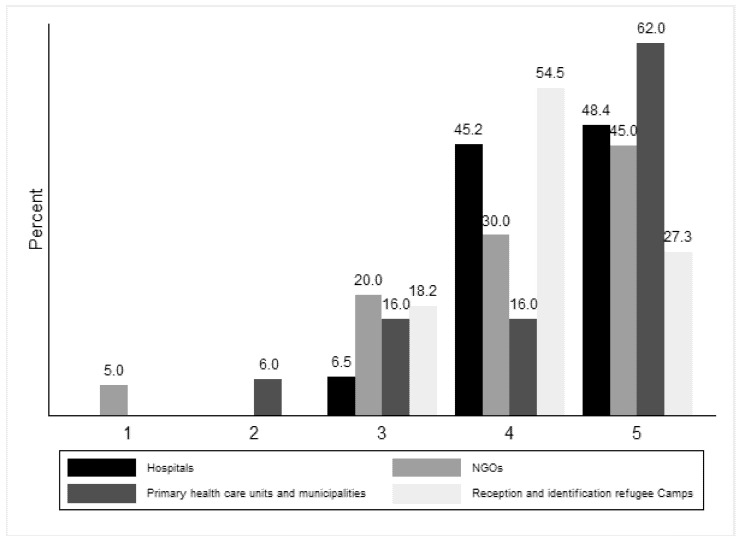
Degree of agreement with phrase “The contact tracing course gave me new ideas on how to improve my communication skills at work” by type of work experience and profile.

The participants’ viewpoint on the statement “ The contact tracing course gave me new ideas on how to improve my communication skills at work” differed depending on the work experience of the participant (p_fisher’s_ = 0.04, Figure 4), with people working in hospitals and in the primary healthcare sector showing a higher level of agreement. Similarly, the course gave these professionals new ideas on how to improve their communication skills in the workplace (Figure 4).

### 3.5. Section 4: Strengths and Weaknesses of the Training Course

The course participants were asked to identify some strengths and to point out ways to improve the contact tracing course. The vast majority considered the course to be extremely well organised, helpful in presenting information on the COVID-19 disease, contact tracing and the skills required to make this process successful. Some participants stated they would like the course to contain more detailed information on the methods of treatment of the disease. They also stressed the fact that the course provides very concrete guidance on how to approach the contact tracing strategy. Many participants, especially health professionals, greatly appreciated the module on the communication skills for contact tracing, because these are competencies that are not covered in their undergraduate degrees, yet communication is an inherent component of their work as they need to be able to communicate efficiently with their patients. As such the course was very effective in increasing their efficiency in their everyday work. Many participants felt that the course was a good starting point for establishing a trained workforce of contact tracers, who could then be employed to support national contact tracing activities and to thereby increase the effectiveness of the pandemic management.

Suggestions for improvement by some participants, especially the non-health professionals, refer to the duration of the course, as they would like it to be slower paced in-order to digest the information presented. Some participants expressed the wish to have more time for discussion and to have the option to discuss some case studies closer to the local context (i.e., refugees, migrants to discuss). Another suggestion was to allow more time for the role playing of scenarios among the course participants in order to better illustrate communication techniques. Moreover, some participants demanded more information on the available social support network possibilities for vulnerable people during quarantine or isolation in addition to the contact tracing activities.

Evaluation results-feedback from the trainers:

With regards to the evaluation of the course from the trainers, a qualitative approach was used. A set of four questions summarising the key points of the evaluation was discussed among the trainers. The answers to these questions provided feedback on various aspects of the contact tracing course, such as its suitability, its effectiveness, its capacity for sustainability and the satisfaction enjoyed by trainees. A total of seven trainers participated in the evaluation.

#### 3.5.1. Suitability: Comment on the Content of the Training Module, the Duration of the Course and the Quiz Questions

Overall, the course was evaluated as being of adequate duration; one trainer suggested to shorten the time of the presentations in order to allow for more time for discussion with the trainees. Several trainers suggested the idea to adapt/modify the presentations in an effort to match the ability level of the trainees. For example, in cases where the trainees have no health education background or belong to a specific population group, infographics in place of text could be available in order to achieve better clarity and understanding. Overall, the course had a satisfactory flow and covered all of the thematic areas that apply to contact tracing. There was a consensus that most of the quiz questions were too easy, but 1–2 were a bit confusing and unclearly phrased. A suggestion was made to add some more challenging questions to prompt in-depth discussion.

#### 3.5.2. Effectiveness: Discuss Your Personal Experience from the Training (e.g., Modules That Generated Questions from the Trainees, Parts That Required Clarifications)

Some trainers identified that the definition of “a close contact “proved to be difficult to comprehend in certain case scenarios, especially for those with limited clinical experience. Clarifications were also required regarding the advice given for the quarantine of close contacts. Similarly, it was necessary to explain the difference between isolation and quarantine (a distinction that is not often made in the Greek language). All of the modules were of interest to the course participants, with a special focus on the mode of transmission, the concept of viral load, the practical issues around quarantine and isolation, as well as the communication techniques. The section on communication methods and the ethical issues around the contact tracing process were highly well received by the course participants. The interest was also very high among the health professionals who attended the course as trainees, as the communication modules gave them guidance and support on how to communicate with their patients in their work settings; a skill which they lack as it is not covered in their undergraduate course curriculum.

#### 3.5.3. Satisfaction: Indicate the Strong Points and Make Suggestions for Improvement

The strong points identified and the suggestions for improvement by the trainees are summarised in the following Table 1.

#### 3.5.4. Sustainability: Discuss the Potential of the Course for Continuation in Greece and Identify Potential Obstacles in Its Implementation

Overall, there is great potential for the continuation of the course in Greece, given the anticipated surges in cases and localised outbreaks. There is a general view that this activity can strengthen the existing epidemiological surveillance system and public health service in the country and can make a substantial contribution to the effectiveness of transmission control and management of the epidemic. The engagement of local stakeholders is essential in order to ensure smooth operation of the contact tracing activities and support, as well as linkage with Primary Healthcare units and local municipalities. The daily follow up suggested by the contact tracing activity provides great added value. It has the potential to address the needs of quarantined and isolated individuals and it increases the chances for effective control of the spread of the disease. Moreover, it has the potential to create a pool of skilled contact tracers, who can expand and greatly improve the contact tracing coverage in all regions of the country. As such, efforts have been initiated to link with the civil protection service and to support the contact tracing activities with the professionals trained through our courses.

## 4. Discussion

Contact tracing is a critical pillar required for a robust and effective COVID-19 response, coupled with vaccination campaigns, social distancing, use of masks, robust screening and diagnostic testing, access to treatment services and social and financial support needed for self-isolation and quarantine of positive cases and their close contacts [9] [20,21]. Trust in public health authorities and structures, health literacy and the ability of people to self-isolate or fully quarantine all have a direct impact on its effectiveness [22,23,24].

Although contact tracing is a key element of a country’s response, investment in contact tracing programmes is essential in order to create a meaningful impact at the local, regional and national levels in breaking chains of transmission. Furthermore, the role of communities participating in the full process of contact tracing is paramount. WHO’s most recent guidance on contact tracing has focused on providing best practice principles for community engagement—including the participation and voices of vulnerable populations in contact tracing efforts—and how such approaches can be operationalised, monitored and measured as part of any community-centred contact tracing strategy [20].

The use of technology in the form of digital applications, platforms and artificial intelligence systems has been proposed as a possible strategy to increase the coverage and effectiveness of contact tracing [25,26,27,28,29,30]. However, issues around privacy, administrative organisation, legal, ethical and cultural considerations have emerged as hurdles to the wider use of technological platforms that could contribute to the effectiveness of contact tracing services [31,32,33,34,35,36]. In addition, the real impact and effectiveness of various technological approaches has not been vigorously evaluated and more research is needed [37,38].

The ability to quickly expand quality services to meet demand has emerged as a problem in need of innovative solutions that will be crucial in the current, but also possible future, pandemics. Different models have been employed to address this need, from the use of digital technology to recruitment of furloughed staff, health sciences students and volunteer led services in the US and elsewhere [39,40,41,42,43,44,45].

Our contract tracing course was positively evaluated by the majority of participants who declared a high degree of satisfaction around their knowledge acquisition about COVID-19, contact tracing strategies and new competencies in communication skills with vulnerable population groups. A substantial number of the participants found the course material to be useful and applicable in their work contexts and expressed the wish to suggest the course to their colleagues. The suggestion to expand the course with more practical training and the presentation of case studies to allow for further understanding of the contact tracing procedure has been noted.

Greece, at the national level, has not yet provided a formal evaluation of its contact tracing programme to date. Such an evaluation could be used to compare with our findings. As stated in the ECDC document on Monitoring and evaluation framework for COVID-19 response activities in the EU/EE and the UK: “ECDC and WHO encourage countries to monitor the effectiveness of their contact tracing operations in order to identify where coverage or timeliness needs to be improved” [46]. However, we recognise the need for further content validity for the developed questionnaire, and we recommend focus group discussions and pre-testing to be utilised in the development of a second round of this questionnaire. As the COVID-19 pandemic is an emergency condition, evolving quickly and with conditions changing rapidly, quasi scientific evaluation approaches are often implemented in various settings (e.g., the evaluation of virus spread in mass gatherings and in large sporting events (UK’s Events Research Programme)).

The work presented in this paper aims to capture the potential for providing skills to professionals working with hard-to-reach populations that can support the optimisation of contact tracing efforts during a public health crisis, as is the case with COVID-19. Our work highlights the high level of satisfaction of the training from professionals working with vulnerable groups, as it is evident that such opportunities are limited, and in reality, lacking. Through the provision of such relevant training and the information on satisfaction, usefulness at work and sustainability, we identify the need to include vulnerable population groups in national disease prevention plans and for professionals to be trained accordingly. During the current pandemic, surveillance, monitoring of the virus spread and contact tracing in vulnerable groups was very limited [47]. Our next steps are to use the successful completion of the evaluation of this training course to advocate for further inclusion of trained professionals in the national contact tracing activities and to monitor such an implementation.

As there has been no nationally coordinated activity among hard-to-reach communities, we have no actual evidence on how effective the contact tracing training could be in such populations residing in Greece. Nonetheless, our evaluation of the training curriculum suggests that the inclusion of such trained professionals in the national contact tracing efforts would be of a public health advantage to health authorities and in pandemic control. Our present work will serve as an advocacy tool to create a network of trained contact tracers and to incorporate them in the national planning strategy for contact tracing.

The evidence that contact tracing programmes for vulnerable populations are positively evaluated by health professionals will help to safeguard the health of these communities in times of health crises and will provide the basis for their incorporation in nationally organised activities.

Modules on communication skills, cultural competence, social support services and ethical issues were greatly appreciated by our trainees, namely health professionals working in hospitals or primary health care. Health workers in Greece, evidently, lack any such training in their professional and academic curricula, whereas the majority of trainees stated that the course could be a sustainable with great added value for the society.

## 5. Conclusions

Our contact tracing training intervention has demonstrated that fast-track training of health care and other professionals under ongoing pandemic conditions is both feasible and acceptable. Our intervention could serve as a blueprint for national health authorities aiming to increase deployment capacities as the pandemic evolves, particularly in the context of the Delta-variant and the most recent surges in cases and transmission. Thus, we strongly recommend national health authorities to consider adopting and scaling-up of this successfully pilot-tested training intervention. As vaccination rates for COVID-19 is increasing in countries, and an increase of positive cases is expected, there is a need to identify close contacts early and effectively. Furthermore, our intervention-trained contact tracers can serve as communicators to vulnerable population groups regarding a wider range of health-related strategies, such as the application of sanitary and hygienic protocols [48], or risk perception and behavioural change practices that can help to increase compliance with protective measures [49]. The experience from the COVID-19 pandemic thus far has shown the importance of primary prevention measures in mitigating the effects of communicable diseases. In addition to contact tracing, these primary prevention measures require long-term strategic planning and the strengthening of primary health care services in order to withstand the pressure from epidemic waves [50].

Through this training activity, a network of qualified contract tracers based within the community that they are engaging with can be established, which will strengthen the epidemiological surveillance systems and will contribute to the successful control of the pandemic in Greece, offering a targeted focus on socially vulnerable native and migrant/refugee communities.

There is great sustainability potential for this activity in Greece, which can be ensured through the collaboration with all stakeholders, such as academia, local regional and municipal authorities and the civil society. Notably, the Greek National Public Health Organisation (NPHO) and the General Secretariat for Civil Protection (GSCP) are urged to capitalise on the contact tracing training organised by the postgraduate program of Global Health- Disaster Medicine (Medical School National and Kapodistrian University of Athens-NKUA). Through this training activity, a network of qualified contract tracers can be established which will strengthen epidemiological surveillance systems and will contribute to the successful control of the pandemic in Greece, offering a special and much needed focus on socially vulnerable native, migrant or refugee communities.

A culturally sensitive and ethically acceptable use of digital technologies based on democratic and transparency principles could be co-developed in cooperation with the country’s research academic body and a variety of communities in Greece that could benefit from such interventions [51,52,53]. Acceptance, usefulness, impact and the various effects of CT in time-spatial variations of the epidemic, should be documented and evaluated alongside epidemiological data and social determinants [54,55]. Promotion of a community-based approach can build trust in public health services through inclusion and participation, increasing health literacy. As a result, such an approach could prove critical in the future stages of the pandemic as vaccination and new emerging variants of SARS-CoV-2 pose risks for new outbreaks locally or nationally [56,57,58]. The increased likelihood of future pandemics is a fundamental reason to continue to invest in innovation on contact tracing, alongside more transparent mechanisms on monitoring and evaluating contact tracing in real-time. Placing issues of equity and health disparities in the centre of our strategies is of paramount importance, as both a moral mandate and as a precondition for successful epidemic control and mitigation of health and social impact [59,60].

An academic-based network of highly trained contact tracing professionals and volunteers, under robust mentorship in linkage with public health services, civil society and affected communities, can have a substantial contribution to the ability of the country to successfully control future COVID-19 outbreaks, as well as other future pandemics.

## Figures and Tables

**Table 1:** Strong points and suggestions for the course by the trainees.

Strong Points of the Training Course, Suggestions for Improvement/Modifications for the Greek Context	
STRONG POINTS	POINTS FOR IMPROVEMENT
Short and clear format of the modules	The inclusion of additional case studies/scenarios taking into account the local context, the characteristics of the target group (e.g., social and cultural parameters, specific barriers, access to healthcare services).
Balanced presentation of the various aspects of the contract tracing process	The provision of options to link with the Primary Health Care services and to provide social support during quarantine.
Appropriate depth and length of the course	The inclusion of scenarios on the contact tracing practices of asymptomatic cases with a positive test (molecular or rapid test).
Availability of quiz questions	Some additional focus on ethical/legal issues concerning contact tracing in population groups residing in special settings such as on the islands, in refugee camps, and in cruise ships.

## Data Availability

The data that support the findings of this study are available from the corresponding author, upon reasonable request.
